# The applications of a commercial gas/liquid separator coupled with an inductively coupled plasma mass spectrometer

**DOI:** 10.1155/S146392469200004X

**Published:** 1992

**Authors:** Peter Hitchen, Robert Hutton, Christopher Tye

**Affiliations:** VG Elemental Ion Path Road Three Cheshire Winsford CW7 3BX UK

## Abstract

A commercially available hydride generator, with a novel membrane gas-liquid separator, has been coupled to a new ICPMS instrument which itself features many unique design considerations. Little or no optimization of the mass spectrometer or ionization source was required to obtain excellent analytical data; and a variety of matrices have been analysed.

The elements As and Se are usually used to demonstrate the effectiveness of a hydride generation system, and these are of particular importance, bearing in mind potential Ar molecular overlaps with isotopes of interest. The flexibility of the hydride generation ICP-MS system is highlighted, with the inclusion of analytical figures of merit for the elements Sn, Sb, Ge and Hg, as well as As and Se. Data obtained by ‘standard’ pneumatic nebulization on the ICP-MS is compared with that obtained with the hydride generator for all of the elements.

Improvements of between 50 and 100 times were gained in measurements of three sigma detection limits for all elements in the determinations, including Hg. Measurements were made on several isotopes for particular elements, and the data is included for the purposes of comparison. Stabilities of between 1 and 2.5% were obtained for 0.5 ppb solutions over 10 min measurement periods, all data is presented without using an internal standard.

Finally, analytical data from seawater standards, spiked with low levels of As and Se and calibrated against aqueous standards, demonstrate excellent recoveries. This is of particular interest bearing in mind the well-documented molecular interferences from high chloride matrices on As and Se analysis.


Journal of Automatic Chemistry, Vol. 14, No. 1 (January–February 1992), pp. 17–23

### The applications of a commercial gas/liquid separator coupled with an inductively coupled plasma mass spectrometer

**Peter Hitchen, Robert Hutton and Christopher Tye**
*VG Elemental, Ion Path, Road Three, Winsford, Cheshire CW7 3BX, UK*

*A commercially available hydride generator, with a novel membrane gas-liquid separator, has been coupled to a new ICP-MS instrument which itself features many unique design considerations. Little or no optimization of the mass spectrometer or ionization source was required to obtain excellent analytical data; and a variety of matrices have been analysed.*

*The elements As and Se are usually used to demonstrate the effectiveness of a hydride generation system, and these are of particular importance, bearing in mind potential Ar molecular overlaps with isotopes of interest. The flexibility of the hydride generation ICP-MS system is highlighted, with the inclusion of analytical figures of merit for the elements Sn, Sb, Ge and Hg, as well as As and Se. Data obtained by 'standard' pneumatic nebulization on the ICP-MS is compared with that obtained with the hydride generator for all of the elements.*

*Improvements of between 50 and 100 times were gained in measurements of three sigma detection limits for all elements in the determinations, including Hg. Measurements were made on several isotopes for particular elements, and the data is included for the purposes of comparison. Stabilities of between 1 and 2·5% were obtained for 0·5 ppb solutions over 10 min measurement periods, all data is presented without using an internal standard.*

*Finally, analytical data from seawater standards, spiked with low levels of As and Se and calibrated against aqueous standards, demonstrate excellent recoveries. This is of particular interest bearing in mind the well-documented molecular interferences from high chloride matrices on As and Se analysis.*

### Introduction

Since the development and commercial introduction [1,2] of inductively coupled plasma mass spectrometry (ICP-MS) in the early 1980s, one of its limiting features has been the occurrence of interferences from certain polyatomic ions [3,4], usually in the region of the spectrum below 80 μ. For example, the determination of As and Se by ICP-MS can be problematic, particularly when samples containing substantial amounts of chloride ions need to be analysed. This is often the case in the field of environmental analysis where saline samples are frequently presented for analysis. The problems associated with chloride are a result of spectral overlap of $^{40}Ar^{35}Cl$ and $^{40}Ar^{37}Cl$ on the isotopes of $^{75}As$ and $^{77}Se$. A number of solutions to these interference problems have been proposed. These include mathematical correction [3], whereby the contribution from the interference is calculated using natural isotopic abundances; chromatographic separation [5,6]; addition of a molecular gas[7] (for example nitrogen); and hydride generation [8].

Hydride generation utilizes the properties of certain elements to form covalent gaseous hydrides which can be generated from acidic solution. Many of the metalloid elements in Groups IVA, VA and VIA of the periodic table (i.e. As, Sb, Se, Bi, Sn, Te, Ge, Hg and Pb) can be determined in this way. These metalloid elements are volatilized by the addition of a reducing agent such as sodium tetrahydroborate to an acidified solution. Other systems have used titanium chloride/magnesium powder and tin chloride/potassium iodide/zinc powder as reducing agents, but the tetrahydroborate method is generally performed because it gives faster hydride formation, higher conversion efficiency, lower blank levels and is simple to use.

The principle of the hydride generation method is:

$$NaBH_4 + 3H_2O + HCl \rightarrow H_3BO_3 + NaCl + 8H$$
$$M^{n+} + 2nH \rightarrow MH_{n\,(g)} + H_{2\,(g)}.$$

The technique has been used with many spectroscopic detectors, i.e. AA, AFS and AES, to enhance sensitivity; a considerable amount of literature is available on the subject.

More recently, hydride generation (HG) ICP-MS [9,10] has been seen as a way of overcoming the limited nebulization and transport efficiency (typically 1–2%) of ICP-MS and thus the potential for an improvement is sensitivity of ~2 orders of magnitude.

However, traditional hydride generation itself is not without its problems and previously its applicability to ICP-MS has been limited, in part because during the hydride reaction excess chloride is carried forward to the plasma through the conventional U-shaped gas-liquid separator. The problem can be reduced by using nitric acid or sulphuric acid media, however, the reduction process is not as efficient.

Recently, a more effective gas-liquid separator has been described [11,12], which utilizes a silicone rubber tubular membrane gas-liquid separator (TMGLS). The TMGLS allows the diffusion of the gas through a tubular microporous membrane, effectively removing any chloride vapour and allowing unambiguous determination of As and Se at masses 75 and 77.

In this paper some applications of a commercially available gas-liquid separator, coupled with a new commercial ICP-MS instrument, are described and the results obtained for the elements As, Se, Sn, Sb, Ge and Hg are shown. The object of the work was to show how

0142–0453/92 $3.00 © 1992 Taylor & Francis Ltd.

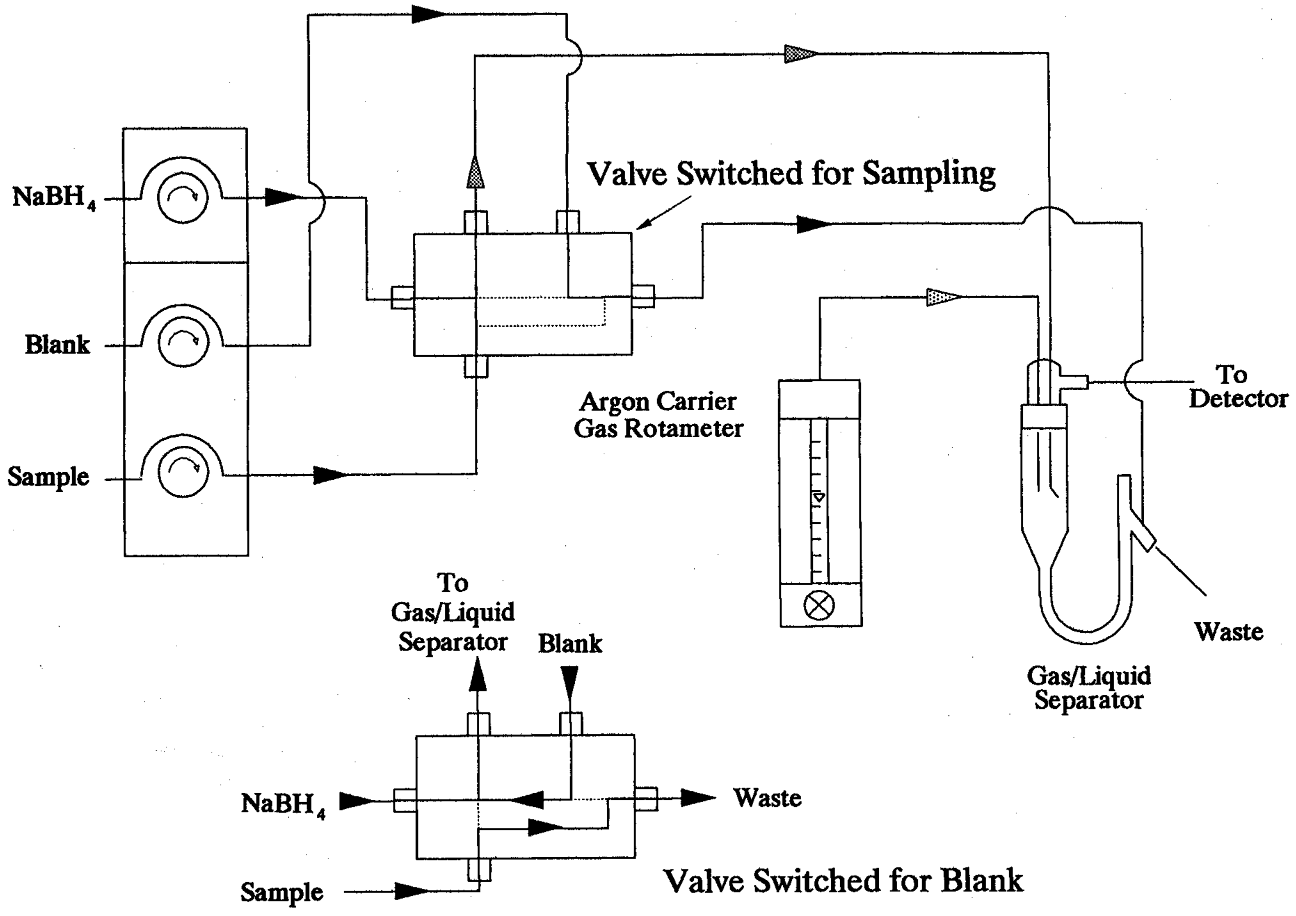

*Figure 1. Schematic of a continuous flow hydride generator.*

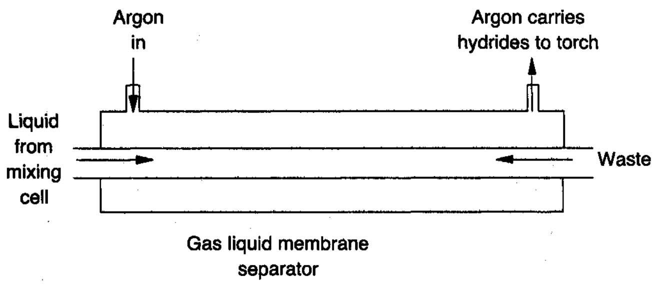

*Figure 2. Schematic of a tubular membrane gas-liquid separator.*

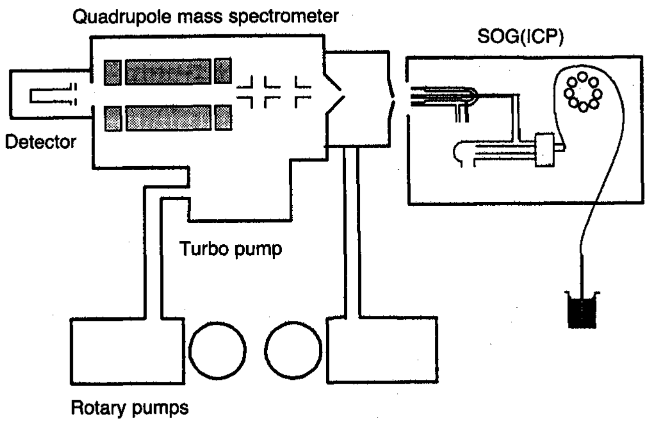

*Figure 3. Schematic of PQe.*

easily hydride generation can be used with ICP-MS, as well as the increase in detection capability that can be achieved.

### Experimental

### *Instrumentation*

A continuous flow hydride generator (PSA 10.003, PS Analytical, Sevenoaks, Kent, UK) was used (figure 1); this was fitted with a tubular membrane gas-liquid separator (see figure 2) in place of the conventional gas-liquid separator.

The ICP-MS instrument used was the PQe (VG Elemental, Winsford, Cheshire, UK). This instrument is different in design from other current commercial ICP-MS instrumentation. It is shown schematically in figure 3 and consists of a free running ICP source (27 MHz frequency). The mass spectrometer is a two-stage system, incorporating only an (atmosphere to vacuum) expansion stage and a high vacuum (quadrupole stage). The system is fabricated of cast aluminium and is pumped by one turbomolecular pump (Edwards Ext 160/500). The quadrupole is designed for this particular instrument and is made of stainless-steel. Since a Faraday detector is used in the instrument, no photon stop is necessary in the ion optics.

*Table 1. Optimal operating conditions.*

| | | |
|---|---|---|
| *ICP-MS* | Forward power | 1500 W |
| | Reflected power | 0 W |
| | Coolant gas flow | 14 l $min^{-1}$ |
| | Auxiliary gas flow | 0·7 l $min^{-1}$ |
| | Carrier gas pressure | 21 psi |
| | Data collection mode | Peak jumping |
| | Analysis time | 9 s/isotope |
| *Hydride generation* | | |
| | Sodium tetrahydroborate solution concentration | 0·1% w/v |
| | Sodium tetrahydroborate solution flow-rate | 3·4 ml $min^{-1}$ |
| | Hydrochloric acid concentration | 2 mol $dm^{-3}$ |
| | Hydrochloric acid/sample flow-rate | 7·5 ml $min^{-1}$ |

Operating and tuning of the instrument is controlled from a Compaq 386s computer utilizing a multi-tasking environment.

The PQe was first fully optimized in its normal configuration for ion lens voltages, nebulizer gas flow rate and quadrupole resolution and pole bias using a standard solution containing elements across the mass range from beryllium to uranium at concentrations of 100 ng $ml^{-1}$.

The nebulizer gas lines were then disconnected and short lengths of Tygon tubing were used to connect on the hydride generator. Since the sample is passed from the hydride generator to the ICP-MS in a gaseous state, it can be connected directly to the end of the plasma torch and so bypasses the normal ICP-MS sample introduction system. Once connected, the optimization was again checked using a 100 ng $ml^{-1}$ arsenic and selenium standard. Forward power was raised to 1500 W and the nebulizer gas pressure reduced from 59 to 21 psi. No further adjustment of the quadrupole or lens settings were required for optimum signal response.

Conditions used are summarized in table 1. All data was obtained using the Peak Jumping mode of acquisition utilized by the PQe.

*Reagents*

All reagents were prepared using deionized water (Elgastat UHP, Elga Ltd, Buckinghamshire, UK). The sodium tetrahydroborate solution was prepared by dissolving 1·0 g of sodium tetrohydroborate (SpectrosoL, BDH) in 1 l of 0·1 mol $dm^{-3}$ sodium hydroxide solution (stabilizer) (Analar, BDH).

The hydrochloric acid solution (2 mol $dm^{-3}$) was prepared by adding 360 ml of concentrated hydrochloric acid (PrimaR grade, Fisons) in 1640 ml of deionized water.

All standard solutions used were prepared by the dilution of the appropriate 1000 mg $l^{-1}$ stock solutions (SpectrosoL, BDH). These dilutions were made with the 2 mol $dm^{-3}$ hydrochloric acid solution.

All reagents and standard solutions were prepared daily, and all volumetric flasks used were previously rinsed in 5% nitric acid (Analar, BDH), left to soak overnight in 2% nitric acid, and then rinsed several times with deionized water.

*Sample preparation*

The seawater and lake water samples analysed were standard solutions obtained from National Research Council of Canada. Each one was acidified with the 2 mol $dm^{-3}$ hydrochloric acid solution prior to analysis. The digested soil samples had been digested using a nitric/perchloric/sulphuric acid mixture prior to dilution.

### Results and discussion

As described earlier, one of the limiting features of ICP-MS has been the occurrence of polyatomic ion interferences, such as $^{40}Ar^{35}Cl$, which precludes the determination of certain isotopic species, in this case $^{75}As$. With the introduction of hydride generation, and, more especially, membrane gas-liquid separation techniques, these problems have been succesfully resolved, such that previously problematic elements such as As and Se can now be analysed with excellent degrees of accuracy.

A further benefit of hydride generation is that the analysis of other elements, such as Sn, Sb, Bi, Sn, Te, Ge, Hg and Pb, that have comparatively high ionization potentials, is also improved because of the increase in sample volume which is passed through to the plasma. The chemical composition of a material analysed by ICP-MS must normally be in a liquid form, which is nebulized by a high-pressure gas flow: Ar in the case of ICP-MS. This nebulization process forms a fine aerosol (very small liquid droplets suspended in a gas) which is carried to the 'analytical region' by the gas. Unfortunately, the nebulization process is inefficient – typically, the nebulizer converts only about 1–2% of the sample volume into useful aerosol. Thus for a flow-rate of 1 ml $min^{-1}$ into the nebulizer, only about 0·02 ml $min^{-1}$ of sample actually reaches the nalytical region. However, with hydride generation the nebulizer is not needed and the rate of sample presentation to the analytical region is changed. The sample is pumped to the hydride reaction zone at the rate of ×7·5 ml $min^{-1}$, in this zone those elements capable of forming gaseous hydrides react and these hydrides are subsequently released from the liquid phase into the argon gas flowing around the membrane separator. The gaseous conversion is approximately 100%. So there is a tremendous increase in the volume of sample which reaches the anlytical region when using a hydride generator. In addition, in the case of the hydride generator, the liquid part of the sample is eliminated from the analytical zone which results in an additional increase in sensitivity (typically by a factor of two to three times).

*Optimization*

There are five parameters which can influence the magnitude of the signal using the HG-ICP-MS technique. These are the concentrations of both the acid and tetrahydroborate solutions, the carrier and auxiliary gas

*Table 2. Stability check on a 0·5 ng ml$^{-1}$ standard.*

| Element | Arsenic | Selenium | | |
|---|---|---|---|---|
| Isotope | $^{75}$As | $^{77}$Se | $^{78}$Se | $^{82}$Se |
| Run 1 | 0·5667 | 0·5294 | 0·5344 | 0·5294 |
| Run 2 | 0·5381 | 0·5160 | 0·5234 | 0·5160 |
| Run 3 | 0·5221 | 0·5095 | 0·5157 | 0·5095 |
| Run 4 | 0·5071 | 0·5036 | 0·5088 | 0·5036 |
| Run 5 | 0·5004 | 0·5040 | 0·5002 | 0·5040 |
| Run 6 | 0·4900 | 0·5024 | 0·4930 | 0·5024 |
| Run 7 | 0·4762 | 0·4862 | 0·4838 | 0·4862 |
| Run 8 | 0·4710 | 0·4828 | 0·4797 | 0·4828 |
| Run 9 | 0·4676 | 0·4836 | 0·4813 | 0·4836 |
| Run 10 | 0·4608 | 0·4825 | 0·4798 | 0·4825 |
| Mean | 0·5000 | 0·5000 | 0·5000 | 0·5000 |
| Sd | 0·0342 | 0·0160 | 0·0198 | 0·0160 |
| % SD | 6·8415 | 3·2018 | 3·9634 | 3·2018 |

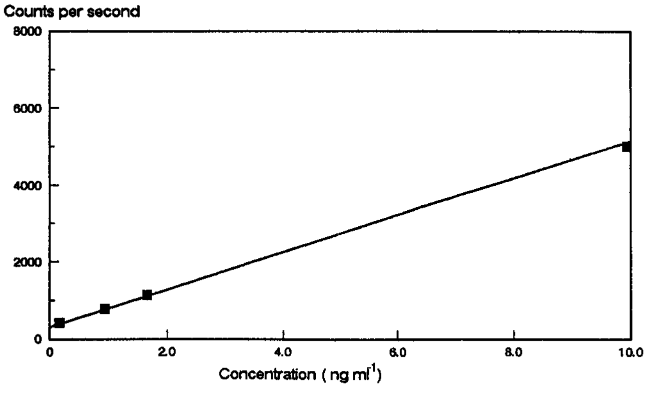

*Figure 4. Calibration graph for $^{75}$As.*

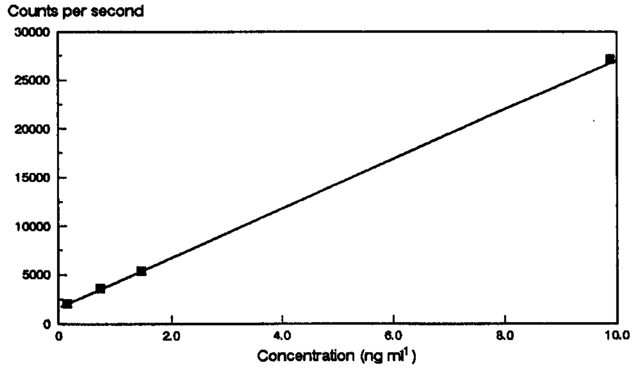

*Figure 5. Calibration graph for $^{78}$Se.*

*Table 3. Comparison of detection limits of HG-PQe with normal pneumatic nebulization (results are in ng ml$^{-1}$).*

| Isotope | PQe + HG | PQe only |
|---|---|---|
| $^{75}$As | 0·0048 | 1·0 |
| $^{77}$Se | 0·0168 | 5·0 |
| $^{78}$Se | 0·0108 | 5·0 |
| $^{82}$Se | 0·0168 | 5·0 |

flows and the forward power. Work has already been carried out to determine the optimum solution concentrations and gas flow rates etc [13]. However, the aim of this work was to demonstrate how easily a hydride generator could be used with a commercially available ICP-MS instrument, and show that, with a minimum of optimization, an improvement in results could be obtained for these elements in varying matrices.

For all the analyses performed, the concentration of the tetrahydroborate and acid solutions were the same as these used by Branch *et al.* [13] in their studies, i.e. the tetrahydroborate was prepared at a concentration of 0·1% w/v in 0·1 mol dm$^{-3}$ sodium hydroxide and the hydrochloric acid prepared at 2 mol dm$^{-3}$. All samples and standards were prepared in 2 mol dm$^{-3}$ hydrochloric acid solution.

The ICP-MS instrument used, the PQe, has three settings for the forward power (low, medium and high) and for this work the power was switched to high (i.e. 1500 W). The neb gas flow was optimized on the $^{75}$As signal; quite a large reduction in pressure from the normal operating flow-rate was required. No adjustment of either the cool or auxiliary gas was made. The ion lenses were tuned for maximum response, again using the $^{75}$As signal, using an automatic tuning facility which is unique to the PQe.

*Table 4. Recoveries from a 10 ng ml$^{-1}$ spike of As and Se in seawaters and estuarine waters (figures in brackets represent the percentage standard deviations).*

| Sample | Dilution | $^{75}$As | $^{77}$Se | $^{78}$Se | $^{83}$Se |
|---|---|---|---|---|---|
| NASS 1 | 20% | 8·55 | 10·51 | 10·62 | 10·49 |
| | | (0·6) | (1·3) | (0·3) | (0·9) |
| NASS 2 | 20% | 8·69 | 10·76 | 10·78 | 10·67 |
| | | (0·4) | (0·8) | (1·2) | (0·8) |
| NASS 2 | 50% | 8·22 | 9·23 | 9·06 | 9·06 |
| | | (0·9) | (2·4) | (1·9) | (1·5) |
| CASS 2 | 20% | 10·86 | 10·60 | 10·72 | 10·76 |
| | | (1·6) | (1·3) | (1·0) | (0·6) |
| SLRS | 20% | 8·67 | 10·20 | 10·17 | 10·27 |
| | | (0·9) | (0·8) | (0·8) | (0·7) |

*Table 5. Analysis of lakewater samples (results are in ng ml$^{-1}$, figures in brackets represent percentage standard deviation).*

| | $^{75}$AS | $^{78}$Se |
|---|---|---|
| Sample 1 | 0·588 (0·6) | 0·093 (1·7) |
| Sample 2 | 0·200 (0·9) | 0·091 (1·0) |
| Sample 3 | 0·434 (0·4) | 0·123 (3.0) |

*Table 6. Analysis of digested river sediments.*

| Element – Selenium | | |
|---|---|---|
| Isotope | Se 78 | Se 82 |
| Run 1 | 415·44 | 423·66 |
| Run 2 | 421·69 | 427·45 |
| Run 3 | 427·43 | 438·45 |
| Mean | 421·18 | 429·85 |
| SD | 5·5100 | 7·6852 |
| % Sd | 1·3082 | 1·7878 |

*Table 7. Comparison of detection limits of PQe, with and without hydride generation (results shown are in ng $ml^{-1}$).*

| Isotope | PQe + HG | PQe only |
|---|---|---|
| $^{121}Sb$ | 0·0033 | 0·5 |
| $^{123}Sb$ | 0·0027 | 0·5 |
| $^{118}Sn$ | 0·0198 | 0·5 |
| $^{120}Sn$ | 0·0216 | 0·5 |
| $^{70}Ge$ | 0·0153 | 1·0 |
| $^{72}Ge$ | 0·0261 | 2·0 |
| $^{73}Ge$ | 0·0282 | 2·0 |
| $^{200}Hg$ | 0·0078 | 0·8 |
| $^{202}Hg$ | 0·0072 | 0·8 |

*Table 8. Stability data obtained for Ge.*

| Isotope | $^{70}Ge$ | $^{73}Ge$ | $^{74}Ge$ |
|---|---|---|---|
| Run 1 | 1·0076 | 1·0058 | 1·0052 |
| Run 2 | 0·9994 | 1·0020 | 1·0027 |
| Run 3 | 1·0006 | 1·0041 | 1·0006 |
| Run 4 | 0·9977 | 0·9992 | 0·9989 |
| Run 5 | 0·9947 | 0·9889 | 0·9925 |
| Mean | 1·0000 | 1·0000 | 1·0000 |
| SD | 0·0048 | 0·0067 | 0·0048 |
| % Sd | 0·4765 | 0·6659 | 0·4790 |

*Table 9. Stability data obtained for Sn.*

| Isotope | $^{116}Sn$ | $^{118}Sn$ | $^{120}Sn$ |
|---|---|---|---|
| Run 1 | 0·9761 | 0·9825 | 0·9865 |
| Run 2 | 0·9881 | 0·9946 | 0·9944 |
| Run 3 | 1·0052 | 1·0069 | 1·0014 |
| Run 4 | 0·9992 | 1·0035 | 1·0053 |
| Run 5 | 1·0313 | 1·0125 | 1·0124 |
| Mean | 1·0000 | 1·0000 | 1·0000 |
| SD | 0·0207 | 0·0117 | 0·0100 |
| % SD | 2·0737 | 1·1732 | 0·9954 |

*Table 10. Stability data obtained for Sb.*

| Isotope | $^{121}Sb$ | $^{123}Sb$ |
|---|---|---|
| Run 1 | 0·9304 | 0·9576 |
| Run 2 | 1·0056 | 1·0127 |
| Run 3 | 1·0253 | 1·0233 |
| Run 4 | 1·0216 | 1·0194 |
| Run 5 | 1·0198 | 1·0088 |
| Run 6 | 1·0097 | 1·0084 |
| Run 7 | 1·0016 | 0·9985 |
| Run 8 | 1·0002 | 0·9972 |
| Run 9 | 0·9932 | 0·9906 |
| Run 10 | 0·9925 | 0·9834 |
| Mean | 1·0000 | 1·0000 |
| SD | 0·0270 | 0·0194 |
| % SD | 2·7008 | 1·9423 |

*Table 11. Stability data obtained for Hg.*

| Isotope | $^{200}Hg$ | $^{202}Hg$ |
|---|---|---|
| Run 1 | 0·9600 | 0·9588 |
| Run 2 | 0·9734 | 0·9840 |
| Run 3 | 0·9718 | 0·9844 |
| Run 4 | 1·0128 | 0·9956 |
| Run 5 | 1·0040 | 0·9918 |
| Run 6 | 1·0215 | 0·9975 |
| Run 7 | 1·0033 | 1·0228 |
| Run 8 | 1·0228 | 1·0101 |
| Run 9 | 1·0262 | 1·0266 |
| Run 10 | 1·0043 | 1·0284 |
| Mean | 1·0000 | 1·0000 |
| SD | 0·0235 | 0·0222 |
| % SD | 2·3518 | 2·2183 |

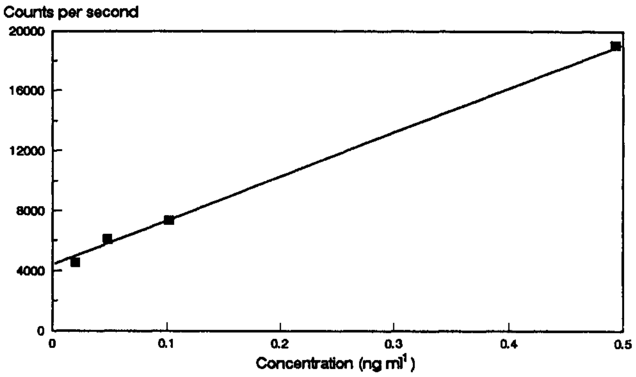

*Figure 6. Calibration graph for $^{74}Ge$.*

*Stability*

In order to be effective for routine analytical work it is essential for the HG-ICP-MS technique to be stable and give reproducible results. In this work, the stability of the instrument configuration was found to be excellent. Table 2 shows stability data for As and Se on a 0·5 ng $ml^{-1}$ standard solution.

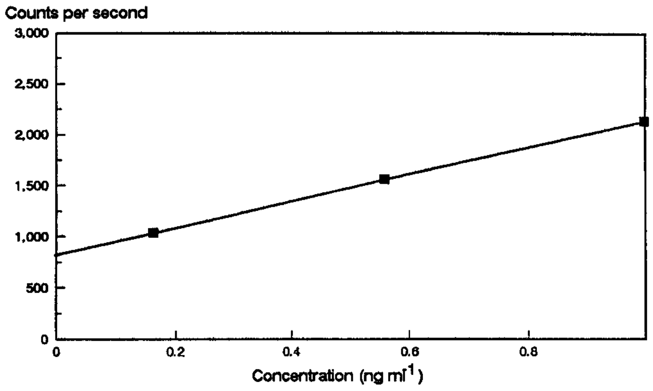

*Figure 7. Calibration graph for $^{120}Sn$.*

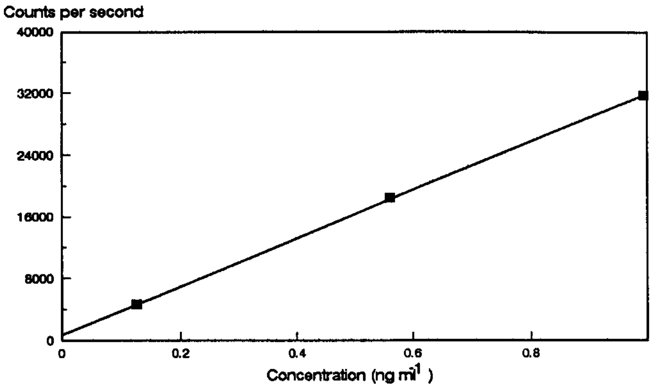

*Figure 8. Calibration graph for $^{121}Sb$.*

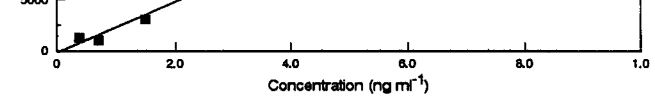

*Figure 9. Calibration graph for $^{202}Hg$.*

*Detection limits*

Once stability had been proved, the next objective was to determine the detection limit of the instrument using this configuration. This was achieved by analysing a blank solution (2 mol $dm^{-3}$ hydrochloric acid) over 10 repeats, and then analysing 10 repeats of a 0·5 ng $ml^{-1}$ standard solution. A calibration graph was then constructed from the standard and the concentration values for the blank solution calculated from this. The detection limits for the elements analysed could then be calculated from 3 sigma of the blank concentrations (10).

Table 3 shows the values obtained for both As and Se. Value for 3 selenium isotopes (77, 78 and 82) have been included to show the excellent reproducibility obtained. Also included in table 3 is a list of detection limits from the PQe without using a hydride generator – a much lower detection limit is achieved using HG-ICP-MS.

*Calibration graphs*

The low detection limits obtained meant that calibration graphs down to sub ng $ml^{-1}$ could easily be achieved.

However, because routine environmental analysis only requires instrument detection capability of *c.* 1 ng $ml^{-1}$, graphs were constructed for As and Se from 0·1 to 10·0 ng $ml^{-1}$. These graphs are shown in figures 4 and 5 respectively.

*Determination of As and Se in seawaters and estuarine waters*

In order to evaluate the validity of the membrane HG-ICP-MS technique on realistic samples, arsenic and selenium determinations were carried out on NASS, CASS and SLRS standard solutions.

Table 4 shows the recoveries from NASS 1, NASS 2, CASS 2 and SLRS samples, diluted to varying degrees and spiked with 10 ng $ml^{-1}$ As and Se. Previous workers [13] have used potassium iodide to reduce arsenite to arsenate prior to hydride generation analysis, but to demonstrate ease of use, this was omitted from this work.

Excellent recoveries were obtained and the low relative standard deviations (typically $<1{\cdot}5\%$ for three repeats) also proves excellent precision. These results once again show the effectiveness of the MGLS in exluding chloride from the ICP-MS instrument, the presence of which would have led to a large positive bias in the analysis.

*Determination of As and Se in lakewater samples and digested river sediments*

To further illustrate the effectiveness of the technique some 'real' samples were analysed – these were samples sent to VG Elemental by prospective customers. These included three lakewater samples and two digested river sediments. The lakewater samples were diluted 9 : 10 with 2 mol $dm^{-3}$ hydrochloric acid prior to analysis, and the river sediment samples diluted 1 : 1, also with 2 mol $dm^{-3}$ (this made a *total dilution* factor of 500 for the sediments).

Table 5 shows the data obtained for $^{75}As$ and $^{78}Se$ for the lakewaters, and table 6 shows the data for $^{78}Se$ and $^{82}Se$ for the sediments. Although we were not informed if the lakewater results reflected the true value, the low standard deviations demonstrate good precision. The values of ~425 ng $ml^{-1}$ obtained for the sediments was in excellent agreement with the customer's value of 417 ng $ml^{-1}$ – this value was the amount to be found in the original sediment, which meant that we needed to find ~0·8 ng $ml^{-1}$ in the diluted sample.

*Further work carried out*

Since the use of the HG-ICP-MS technique was so successful for the analysis of As and Se, it was decided to perform further analyses to establish its effectiveness for other metalloid elements in groups IVA, VA and VIA – namely Sb, Sn, Ge and Hg. These elements were volatilzied and reduced in a similar manner to As and Se, although Ge and Hg are reduced to a lesser degree.

Table 7 shows the detection limits obtained for Sb, Sn, Ge and Hg. Once again there is a dramatic increase in detection capability, compared with the results obtained using the normal pneumatic nebulization of the PQe.

Tables 8 to 11 shows examples of stability data obtained for the four elements when a 1·0 ng $ml^{-1}$ standard was aspirated. Excellent stability was achieved for the 1·0 ng $ml^{-1}$ standard, with all percentage standard deviations (RSDs) falling below 3% for both the fifth and tenth repeats. Calibration graphs for the four elements have also been included to show the good linearity obtained (Figures 6–9).

### Conclusions

The use of a membrane gas-liquid separator with continuous flow hydride generation has been shown to be an effective method for use with ICP-MS. A large increase in the detection capability has been achieved, and linear calibration plots were obtained from 0·1 to over 10 ng $ml^{-1}$ using the PQe. The advantage of the MGLS to dampen pump noise also allows good reproducibility, typically <3%.

The ability of the technique to overcome chloride interferences when determining both arsenic and selenium is obviously a major benefit when analysing samples that have a high chloride content. In addition, its ability to analyse other elements that can be problematic in ICP-MS (i.e. Sn, Sb, Ge and Hg) is a great advantage.

### Acknowledgements

The authors would like to thank Ed McCurdy and also Warren Corns for their help during the compilation of this report. Many thanks also to Peter Stockwell.

### References

1. Gray, A. L., In *Applications of Inductively Coupled Plasma Mass Spectrometry* (Eds. Date, A. R. and Gray, A. L.) (Blackie and Son, Glasgow, 1989), 1.
2. Gray, A. L. and Date, A. R., *Analyst,* **108** (1983), 1033.
3. Munro, S. Ebdon, L. and McWeeney, D. J., *Journal of Analytical Atomic Spectrometry,* **1** (1986), 211.
4. Tan, S. H. and Horlick, G., *Applied Spectroscopy,* **4** (1986), 445.
5. Branch, S., Bancroft, K. C. C., Ebdon, L. and O'Neil, P., *Analytical Proceedings,* **26** (1989), 73.
6. Lyon, T. D. B., Fell, G. S., Hutton, R. C. and Eaton, A. N., *Journal of Analytical Atomic Spectrometry,* **3** (1988), 265.
7. Evans, E. H. and Ebdon, L., *Journal of Analytical Atomic Spectrometry,* **4** (1989), 299.
8. Branch, S., Corns, W. T., Ebdon, L., Hill, S. and O'Neil, P., *Journal of Analytical Atomic Spectrometry,* **6** (1991), 151.
9. Powell, M. J., Boomer, D. W. and McVicars, R. J., *Analytical Chemistry,* **58** (1986), 2684.
10. Dean, J. R., Parry, H. G. M., Massay, R. C. and Ebdon, L., *ICP Information Newsletter,* **15** (1990), 569.
11. Cave, M. R. and Green, K. A., *Atomic Spectroscopy,* **9** (1988), 149.
12. Cave, M. R. and Green, K. A., *Journal of Analytical Atomic Spectroscopy,* **4** (1989), 223.
13. Branch, S., Corns, W. T., Ebdon, L., Hill, S., O'Neil, P., *Journal of Analytical Atomic Spectroscopy,* **6** (1991), 155.

